# A randomized phase II study of docetaxel or pemetrexed with or without the continuation of gefitinib after disease progression in elderly patients with non‐small cell lung cancer harboring *EGFR* mutations (JMTO LC12‐01)

**DOI:** 10.1111/1759-7714.14465

**Published:** 2022-05-13

**Authors:** Kazuhiro Asami, Masahiko Ando, Takashi Nishimura, Takashi Yokoi, Atsuhisa Tamura, Koichi Minato, Masahide Mori, Fumitaka Ogushi, Akiyoshi Yamamoto, Hiroshige Yoshioka, Masaaki Kawahara, Shinji Atagi

**Affiliations:** ^1^ Asami Naika Clinic Tokyo Japan; ^2^ Department of Advanced Medicine Nagoya University Hospital Nagoya Japan; ^3^ Department of Respiratory Medicine Kyoto Katsura Hospital Kyoto Japan; ^4^ Department of Thoracic Oncology Hyogo College of Medicine Hyogo Japan; ^5^ Department of Center for Pulmonary Diseases Tokyo National Hospital Tokyo Japan; ^6^ Department of Respiratory Medicine Gunma Prefectural Cancer Center Gunma Japan; ^7^ Department of Thoracic Oncology National Hospital Organization Osaka Toneyama Medical Center Osaka Japan; ^8^ Department of Respiratory Medicine Kochi National Hospital Kochi Japan; ^9^ Department of Respiratory Medicine Takamatsu Red Cross Hospital Kagawa Japan; ^10^ Department of Thoracic Oncology Kansai Medical University Hospital Osaka Japan; ^11^ Department of Respiratory Medicine KKR Otemae Hospital Osaka Japan; ^12^ Department of Thoracic Oncology National Hospital Organization Kinki‐Chuo Chest Medical Center Osaka Japan

**Keywords:** chemotherapy, elderly, epidermal growth factor receptor mutation, gefitinib, non‐small cell lung cancer

## Abstract

**Background:**

Gefitinib (G) is a recommended molecular‐targeted agent for elderly patients with epidermal growth factor receptor (EGFR)‐mutant non‐small cell lung cancer (NSCLC). Docetaxel (Doc) and pemetrexed (Pem) have similar efficacies, and either is often used as the sole agent during treatment. The efficacy of continuing G after progressive disease (PD) develops has been reported. It remains unclear whether the continuation of G in combination with a single cytotoxic agent beyond PD is beneficial for elderly patients. Here, we conducted a randomized phase II study to assess the efficacy and safety of cytotoxic chemotherapy with G for elderly patients with progressive *EGFR*‐mutant NSCLC.

**Methods:**

Elderly patients with *EGFR*‐mutant NSCLC with PD previously treated with G were enrolled. Patients received Pem 500 mg/m or Doc 60 mg/m every 21 days and were randomly assigned to receive chemotherapy with 250 mg G (G+ Doc/Pem arm) or without G (Doc/Pem arm) until further disease progression or unacceptable toxicity.

**Results:**

This trial was terminated early owing to slow accrual. A group of 22 patients underwent analysis. The primary endpoint, progression‐free survival (PFS), was significantly longer in the G + Doc/Pem arm (median: 1.6 months vs. 5.6 months, hazard ratio = 0.40, 95% CI: 0.16–0.99, *p* = 0.0391). Adverse events ≥ grade 3 were more frequent in the G + Doc/Pem arm (45.5% vs. 90.9%, *p* = 0.032).

**Conclusions:**

Patients on G and Pem or Doc beyond PD showed a longer PFS than those on single‐agent chemotherapy; however, it was associated with increased toxicity.

## Graphical abstract

We conducted a randomized phase II study to assess the efficacy and safety of cytotoxic chemotherapy with gefitinib for elderly patients with progressive *EGFR*‐mutant NSCLC. PFS was significantly longer in the gefitinib with chemotherapy arm (median: 1.6 months vs. 5.6 months, HR = 0.40, 95% CI: 0.16–0.99, *p* = 0.0391). Adverse events ≥ grade 3 were more frequent in the gefitinib with chemotherapy arm (45.5% vs. 90.9%, *p* = 0.032). Based on our results, combination chemotherapy with continuing gefitinib beyond PD may have potential benefit in limited elderly NSCLC without information on acquired resistance to gefitinib.

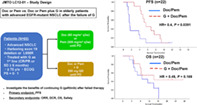

## INTRODUCTION

Lung cancer is the leading cause of cancer death worldwide.^1^, ^2^, ^3^ Approximately 70% of patients with lung cancer patients are diagnosed with advanced non‐small cell lung cancer (NSCLC), and the prevalence and lung cancer mortality have been increasing in Japan.^4^ These increases are more prominent in elderly Japanese patients because of its super‐aging society; elderly patients aged ≥70 years account for 75% of lung cancer deaths.^5^

Docetaxel (Doc) is a third‐generation cytotoxic agent and recommended monotherapy regimen for elderly patients with advanced NSCLC.^6^ Large‐scale trials to demonstrate the efficacy of pemetrexed (Pem) monotherapy in elderly patients with advanced NSCLC have not yet been performed. However, the efficacy of Pem in elderly patients is expected to be similar to that of Doc. Based on the findings of a subset analysis of a phase III study on Pem and Doc^7^ and a review of two randomized studies on Pem,^8^ time to progression, overall survival (OS), and toxicity profiles were favorable for Pem. Therefore, Doc and Pem are both recommended for the treatment of elderly Japanese patients with advanced NSCLC.

Gefitinib (G) is a first‐generation epidermal growth factor receptor‐tyrosine kinase inhibitor (EGFR‐TKI) with a demonstrated strong efficacy in patients harboring active *EGFR* mutations.^9^, ^10^, ^11^ Phase II studies showed the utility and efficacy of G as first‐line therapy for elderly patients with advanced *EGFR*‐mutant NSCLC.^12^, ^13^

Previous studies reported the utility and safety of combination therapy with first‐generation EGFR‐TKIs, such as G and erlotinib, plus Doc or Pem for advanced NSCLC.

Manegold et al. assessed the feasibility of combination therapy with G and Doc in patients with advanced NSCLC.^14^ Fifteen patients each were recruited for different doses of G. The most common observed adverse events (AEs) were consistent with the known profiles of G, and dose‐limiting toxicities were not detected. Therefore, the combination of G and Doc is feasible and acceptable for patients with NSCLC.

Yoshimura et al. assessed the efficacy and toxicity of combination therapy with G and Pem in elderly patients (≥ 70 y) with previously untreated NSCLC and a performance status (PS) of 0–1.^15^ A total of 44 eligible patients were enrolled. The overall response rate (RR) was 29%, with 48% for stable disease (SD). The median progression‐free survival (PFS) and OS were 8 (95% confidence interval [CI]: 6.2–10.6) and 12 months (95% CI: 5.6–17.5) for all patients, respectively. The most common hematological AEs were lymphopenia and anemia, while the most common nonhematological AEs were fatigue, hyperglycemia, and dyspnea. These findings suggested that the combination of one cytotoxic drug plus G is an acceptable regimen for elderly patients with advanced NSCLC.

Although the efficacy of G for advanced *EGFR*‐mutant NSCLC has been demonstrated, most patients relapse after approximately 1 year. Some *EGFR*‐mutant NSCLCs acquire resistance to G and show accelerated disease progression after its discontinuation.^16^, ^17^ The continuation of G combined with a single cytotoxic drug to reduce the risk of accelerated disease progression is a promising strategy for elderly patients with *EGFR*‐mutant NSCLC.

Therefore, we conducted a prospective phase II study to evaluate the efficacy and safety of the continuation of G combined with Doc or Pem in elderly patients with *EGFR*‐mutant NSCLC.

## METHODS

### Inclusion criteria

We enrolled patients aged ≥70 years with adequate major organ functions and cytologically‐ or histologically‐confirmed active *EGFR*‐mutant stage IIIB or IV NSCLC, including the exon 19 deletion mutation or L858R, after the confirmation of radiological disease progression during G therapy of at least 3 months. They were required to have a measurable or assessable disease as defined by the Response Evaluation Criteria in Solid Tumors (RECIST) version 1.1 and a PS of 0 or 1 by the Eastern Cooperative Oncology Group (ECOG) criteria. Patients with a complete response (CR), partial response (PR), or SD for >6 months while being treated with G were eligible.

### Exclusion criteria

We excluded patients who were advised to switch to platinum‐doublet chemotherapy or patients with any of the following: a history of interstitial pneumonia and pre‐existing idiopathic pulmonary fibrosis pneumonia, active infectious disease, and uncontrolled pleural or cardiac effusion.

The study protocol was approved by each institution's ethics review board, and the study was conducted in accordance with the Declaration of Helsinki and Good Clinical Practice guidelines. The study was registered with University Hospital Medical Information Network Clinical Trials Registry (ID, UMIN 000007765). All patients provided written informed consent before enrollment.

### Study design

**FIGURE 1:**
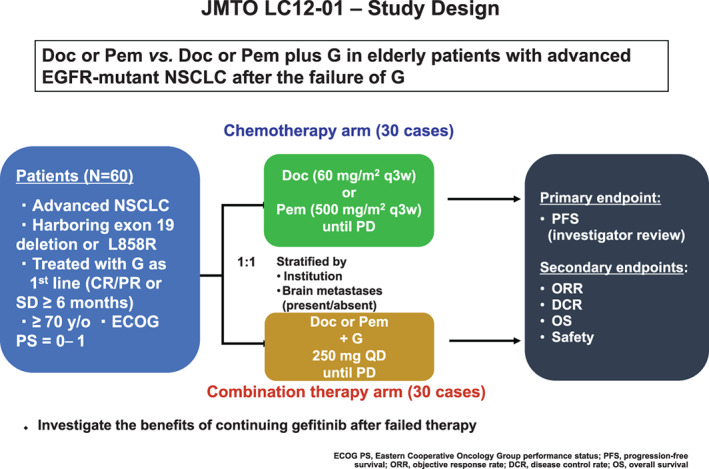
Treatment scheme of JMTO LC12‐01. Eligible patients were randomized in a 1:1 ratio to receive docetaxel (Doc) or pemetrexed (Pem) therapy alone (chemotherapy arm) or the continuation of gefitinib (G) plus Doc or Pem (combination therapy arm). Doc, docetaxel; G, gefitinib; Pem, pemetrexed; NSCLC, non‐small cell lung cancer; ECOG PS, Eastern Cooperative Oncology Group performance status; PFS, progression‐free survival; ORR, objective response rate; DCR, disease control rate; OS, overall survival; CR, complete response; PR, partial response; SD, stable disease

The study design is shown in Figure 1. Eligible patients were randomized in a 1:1 ratio to receive Doc or Pem alone (Doc/Pem arm) or the continuation of G plus Doc or Pem (G + Doc/Pem arm). Patients assigned to the Doc/Pem arm received Doc (60 mg/m^2^ intravenously [IV] every 3 weeks) or Pem (500 mg/m^2^ IV every 3 weeks, with premedication, including folic acid and vitamin B_12_, according to the pemetrexed label), while those assigned to the G + Doc/Pem arm continued to receive G with Doc (60 mg/m^2^ IV every 3 weeks) or Pem (500 mg/m^2^ IV every 3 weeks with the same premedication). G (250 mg daily or as tolerated) was continued on the administration schedule that had been maintained for at least three months before registration. Treatment continued until disease progression, unacceptable toxicity, or other study discontinuation criteria were met.

### Treatment assessments

The primary endpoint was PFS. The secondary endpoints were response rates (CR plus PR), disease control rates (DCR: CR plus PR plus SD), OS, and toxicity profiles. PFS was defined as the period between the date of randomization to the date of objective progression or death. OS was defined as the period between the date of randomization to the date of death due to any cause.

SD had to meet the criteria of SD at least once >6 weeks after randomization. All enrolled patients were evaluated for safety and assessed for toxicity before each cycle using the Common Terminology Criteria for Adverse Events Version 4.03. They underwent full‐body computed tomography every 6 weeks until PD was achieved, and they were examined using RECIST version 1.1.

### Statistical analysis

With PFS as the primary endpoint, Shoenfeld's formula^18^ was used to calculate the sample size for this study. The PFS of Doc and Pem in previous studies was approximately 2.0 months as second‐line therapy and 5.5 months as first‐line therapy for elderly patients with NSCLC.^6^, ^19^ A randomized phase III trial of Pem versus Doc in previously treated patients with advanced NSCLC reported the same PFS of 2.9 months for each treatment arm.^19^ The PFS of patients who received epidermal growth factor receptor tyrosine kinase inhibitors (EGFR‐TKIs) plus chemotherapy was 7.0 and 8.0 months, respectively for patients who received EGFR‐TKIs plus Pem.^14^, ^15^ We set a 6‐month PFS as a primary endpoint, which is two months longer than that for chemotherapy including Doc or Pem alone, as suggested in the aforementioned trials. This study was designed to detect a prolonged PFS with G + Doc/Pem combination therapy with an 80% power using the log‐rank test with a one‐sided α of 5%, an accrual period of 12 months, and a follow‐up period of 24 months. The estimated minimum accrual was 28 cases. Assuming a patient dropout rate of approximately 10%, the sample size was 30 patients in each arm. Central randomization was performed using the minimization method of balancing institution and brain metastasis (yes or no).

PFS and OS were estimated using the Kaplan–Meier method, and the *p*‐value for differences between curves was calculated using the log‐rank test. Hazard ratios and associated 95% CIs between the treatment arms were calculated. Differences in RRs and frequency of AEs, and the associated 95% CIs were calculated and compared with Fisher's exact test. A *p*‐value <0.05 was considered statistically significant. Statistical analyses were performed using the software SAS version 9.2 (SAS Institute).

## RESULTS

### Patient characteristics

This study was closed in early July 2016 because of poor accrual. Between April 2012 and August 2015, a preliminary analysis involving 22 patients randomly assigned to receive Doc or Pem alone or the continuation of G + Doc/Pem was conducted. The patient and disease baseline characteristics are shown in Table 1. All registered patients received at least one cycle of chemotherapy. Two patients in each arm failed to meet the inclusion criteria. All the patients had adenocarcinomas and activated *EGFR* mutations, including exon 19 deletion and L858R.

**TABLE 1:** Patient characteristics

Characteristic		No. of patients (%)	
		Chemotherapy^a^ arm (*n* = 11)	Gefitinib plus chemotherapy^a^ arm (*n* = 11)
Median age, years (range)		76 (72–84)	78 (70–86)
Sex			
	Male	1 (10)	1 (10)
	Female	10 (90)	10 (90)
Performance status (ECOG)			
	0	6 (55)	4 (36)
	1	5 (45)	7 (64)
Stage			
	IV	11 (100)	11^b^ (100)
Histology			
	Adenocarcinoma	11 (100)	11 (100)
Smoking history			
Never smoker		9 (82)	8 (73)
Ex‐smoker		2 (18)	3 (27)
*EGFR* mutation status			
	Exon 19 deletion	5 (45)	6 (55)
	Exon 18	6 (55)	5 (45)
Brain metastasis			
		3 (27)	3 (27)
Responses to gefitinib			
	PR	7 (64)	9 (82)
	SD	4 (36)	2 (18)
Median duration of gefitinib administration (months), range			
		10.4 (4.8—66.3)	9.6 (7.6–37.1)
Administration period of gefitinib (months)			
	Once daily	7 (64)	9 (82)
	Others	4^d^ (36)	2^c^ (18)
Chemotherapy agent			
	Docetaxel	3 (27)	5 (45)
	Pemetrexed	8 (73)	6 (55)

Abbreviation: ECOG, Eastern Cooperative Oncology Group; EGFR, epidermal growth factor receptor, PR, partial response; SD, stable disease.

^a^ Docetaxel or pemetrexed.

^b^ Postoperative recurrences occurred in three patients.

^c^ Alternate‐day administration (1 patient) and once every three days (1 patient).

^d^ Alternate‐day administration (2 patients), once every three days (1 patient), and twice a week (1 patient).

Three patients in the Doc/Pem arm and five in the G + Doc/Pem arm received Doc, while eight patients in the Doc/Pem arm and six in the G + Doc/Pem arm received Pem. More than 50% of enrolled patients were administered G once daily and all patients continued G on the same schedule as before enrollment in each treatment arm.

Ten patients in the Doc/Pem arm and four in the G + Doc/Pem arm discontinued the protocol treatment due to disease progression, which was observed after one cycle of chemotherapy in two out of the 10 patients (18%) in the Doc/Pem arm. Nonhematological AEs did not resolve until the next treatment cycle in one patient in the Doc/Pem arm. In the G + Doc/Pem arm, protocol therapy was discontinued in seven patients because of their requests or judgments by the attending physician based on a reduced PS due to nonhematological AEs (fatigue, diarrhea, and anorexia).

The median number of chemotherapy treatment cycles was two for each chemotherapy group in the Doc/Pem arm, six for the Doc group, and four for the pemetrexed group in the G + Doc/Pem arm (Table 2).

**TABLE 2:** Treatment agents and their administration

		Chemotherapy^a^ arm	Gefitinib plus chemotherapy^a^ arm
		(*n* = 11)	(*n* = 11)
Docetaxel			
	Median treatment cycle (range)	2 (1–2)	6 (1–9)
Pemetrexed			
	Median treatment cycle (range)	2 (1–18)	4 (1–10)
Duration period of gefitinib			
	Median months (range)	NA	2.9 (0.2–9.2)

Abbreviation: NA, not applicable.

^a^ Docetaxel or pemetrexed.

### Efficacy

**FIGURE 2:**
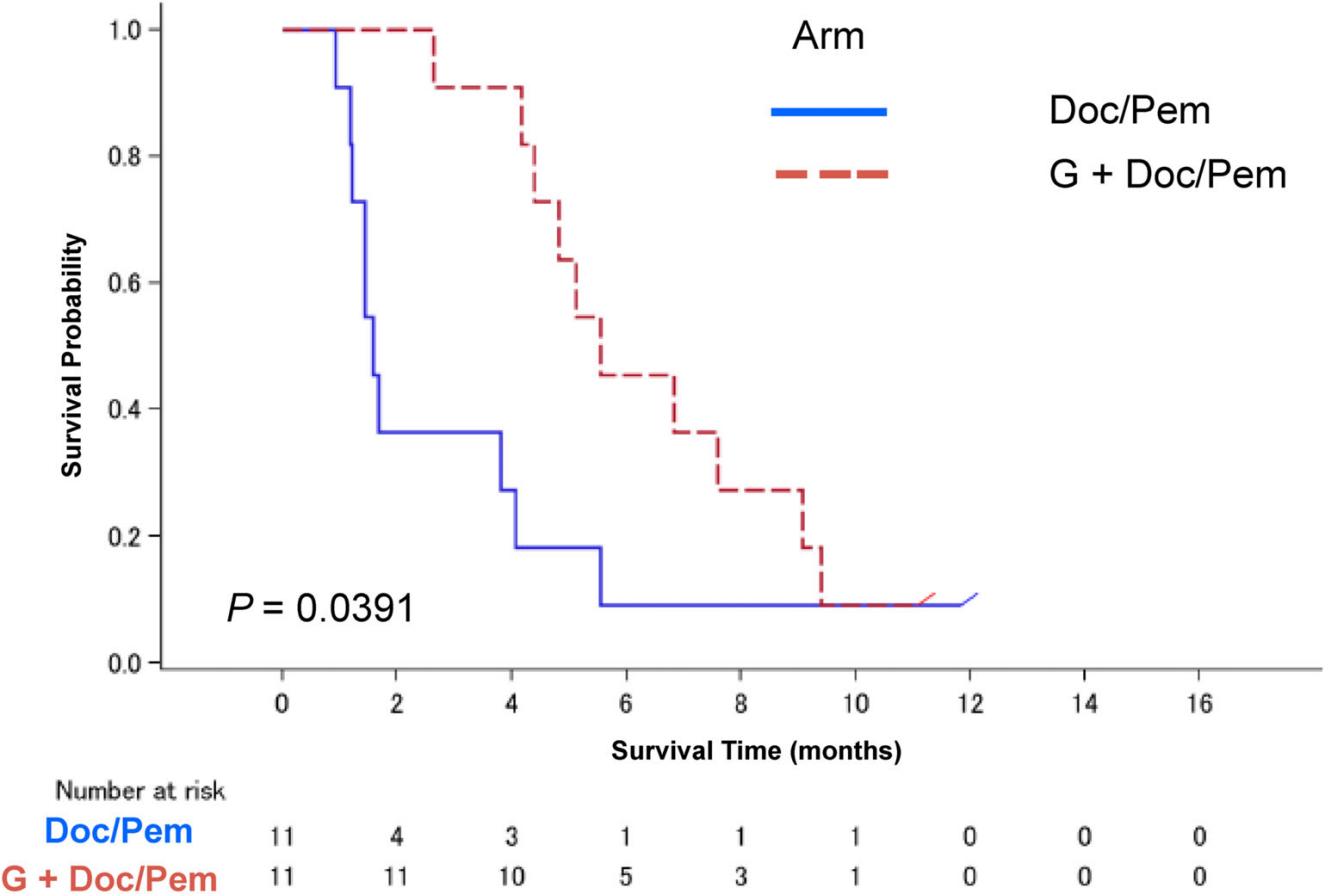
Kaplan–Meier plot of progression‐free survival (PFS) (*n* = 22). The median PFS was 1.6 months (95% CI: 1.2–4.1 months) in the Doc/Pem arm and 5.6 months (95% CI: 4.2–9.1) in the G + Doc/Pem arm. A significant difference was observed between the two arms (HR = 0.4, 95% CI: 0.16–0.99, p* = 0.0391). Doc, docetaxel; G, gefitinib; Pem, pemetrexed; PFS, progression‐free survival

The median follow‐up time was 8.8 months. The median PFS and OS were 1.6 months (95% CI: 1.2–4.1) and 8.6 months (95% CI: 4.6–16.3) in the Doc/Pem arm and 5.6 months (95% CI: 4.2–9.1) and 14.1 months (95% CI: 5.1‐not reached) in the G + Doc/Pem arm (Figure 2), respectively. A significant difference was observed in PFS (HR = 0.40, 95% CI: 0.16–0.99, *p* = 0.0391), but not in OS (HR = 0.49, 95% CI: 0.17–1.39, *p* = 0.169).

RR and DCR were 0% (95% CI: 0.0–28.5) and 27.3% (95% CI: 6.0–61.0) in the Doc/Pem arm, and 18.2% (95% CI: 2.3–51.8) and 54.5% (95% CI: 23.4–83.3) in the G + Doc/Pem arm. There were no significant differences in RR (*p* = 0.476) or DCR (*p* = 0.387) between the two arms (Table 3).

**TABLE 3:** Objective response

	Chemotherapy^a^ arm (*n* = 11)	Gefitinib plus chemotherapy^a^ arm (*n* = 11)	*p*‐value^c^
CR	0	0	
PR	0	2 (18.2%)	
SD^b^	3 (27.3%)	4 (36.4%)	
PD	7 (63.6%)	1 (9.1%)	
NA	1 (9.1%)	4 (36.4%)	
Overall response rate (95% CI)	0% (0.0–28.5)	18.2% (2.3–51.8)	0.476
Disease control rate	27.3% (6.0–61.0)	54.5% (23.4–83.3)	0.387

Abbreviation: CR, complete response; PR, partial response; SD, stable disease; PD, progressive disease; NA, not applicable; CI, confidence interval; NA, not evaluated.

^a^ Docetaxel or pemetrexed.

^b^ Stable disease was confirmed and sustained for 6 weeks or longer.

^c^ Fisher's exact test was used, and *p* < 0.05 was significant.

### Toxicity

**FIGURE 3:**
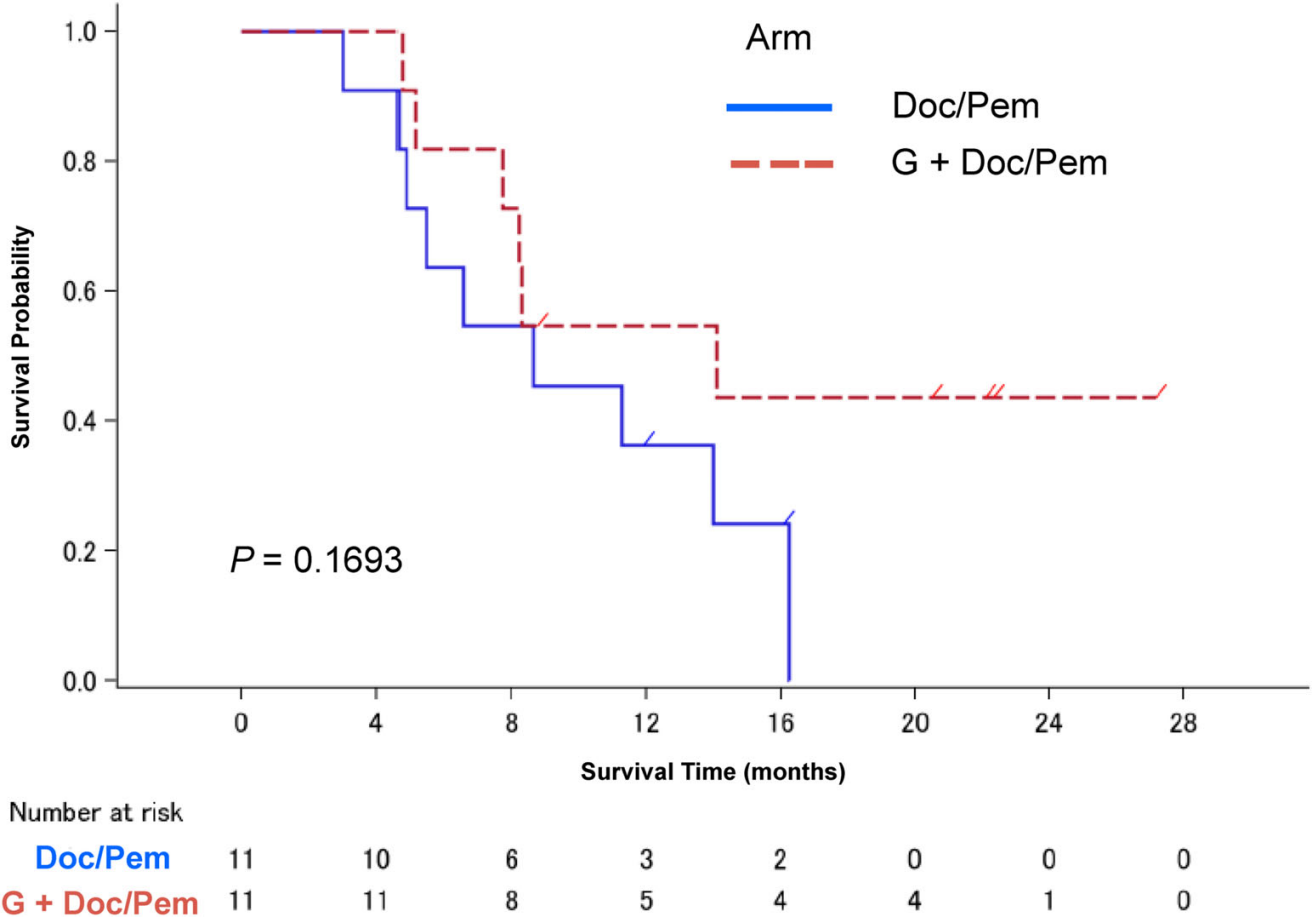
Kaplan–Meier plot of overall survival (OS) (*n* = 22). The median OS was 8.6 months (95% CI: 4.6–16.3 months) in the Doc/Pem arm and 14.1 months (95% CI: 5.1–not reached) in the G + Doc/Pem arm. No significant difference was observed between the two arms (HR = 0.49, 95% CI: 0.17–1.39, p* = 0.169). *p < 0.05 was significant. Doc, docetaxel; G, gefitinib; Pem, pemetrexed

Toxicity assessments were conducted on all treated patients (*n* = 22). Severe toxicities (≥ grade 3) were observed in five patients (45.5%): leukopenia and neutropenia in three (27.3%) and four patients (36.4%), respectively, in the Doc/Pem arm. Lymph node infection, a grade 3 nonhematological toxicity, occurred in one patient. The frequency of severe hematological or nonhematological toxicities (≥ grade 3) was significantly higher in the G + Doc/Pem arm (90.9% vs. 45.5%, *p* = 0.032). The severe toxicities (≥ grade 3) in the G + Doc/Pem arm included leukopenia and neutropenia in 7 (63.6%) patients each and febrile neutropenia in two (18.2%) patients. Grade 3 toxicities, that is, anorexia and fatigue were observed in three and four patients (36.44%), respectively (Table 4). Three patients in each arm needed chemotherapy dose reductions due to treatment toxicity. Among patients receiving Doc, two out of three (66.7%) were in the Doc/Pem arm, while three out of five (60%) were in the G + Doc/Pem arm. One of the eight patients (12.5%) receiving Pem was in the Doc/Pem arm (Table 5). There were no treatment‐related deaths in either arm (Figure 3).

**TABLE 4:** Overall summary of adverse events

		Chemotherapy^a^ arm (*n* = 11)	Gefitinib plus chemotherapy^a^ arm (*n* = 11)	*p*‐value^b^
Severe AEs (≥ grade 3)		5 (45.5%)	10 (90.9%)	0.032
AEs led to dose reduction	Docetaxel	*n* = 3	*n* = 5	
		2 (66.7%)	3 (60.0%)	0.714
	Pemetrexed	*n* = 8	*n* = 6	
		1 (12.5%)	0 (0.0%)	0.571

Abbreviation: AE, adverse event; NA, not applicable.

^a^ Docetaxel or pemetrexed.

^b^ Fisher's exact test was used, and *p* < 0.05 was significant.

**TABLE 5:** Toxicities (≥ grade 3)

Toxicity	Chemotherapy^a^ arm (*n* = 11)		Gefitinib plus chemotherapy^a^ arm (*n* = 11)	
	Docetaxel *n* = 3 (%)	Pemetrexed *n* = 8 (%)	Docetaxel *n* = 5 (%)	Pemetrexed *n* = 6 (%)
Leucopenia	3 (100.0)	0 (0.0)	5 (100.0)	2 (33.3)
Neutropenia	3 (100.0)	1 (12.5)	3 (60.0)	4 (66.7)
Febrile neutropenia	1 (33.3)	0 (0.0)	2 (40.0)	0 (0.0)
Thrombopenia	0 (0.0)	0 (0.0)	0 (0.0)	1 (16.7)
Anemia	0 (0.0)	0 (0.0)	1 (20.0)	1 (16.7)
Anorexia	0 (0.0)	0 (0.0)	3 (60.0)	0 (0.0)
Fatigue	0 (0.0)	0 (0.0)	3 (60.0)	1 (16.7)
Diarrhea	0 (0.0)	0 (0.0)	1 (20%)	0 (0.0)
Infection	1 (33.3)	0 (0.0)	0 (0.0)	1 (16.7)
Others^b^	1 (33.3)	1 (12.5)	2 (40%)	2 (33.3)

^a^ Docetaxel or pemetrexed.

^b^ Lymphopenia, elevated levels of alanine aminotransferase, hypoalbuminemia, and electrolyte abnormality.

## DISCUSSION

This is the first randomized phase II study to assess the efficacy of continuing G with chemotherapy beyond PD in elderly patients with advanced *EGFR*‐mutant NSCLC. However, this study ended in early July 2016 due to slow enrollment; the calculated sample size was not attained.

In this preliminary study, PFS was significantly longer in the G + Doc/Pem arm than in the Doc/Pem arm (HR = 0.40, 95% CI: 0.16–0.99, *p* = 0.0391). The median OS was approximately 14 months in the G + Doc/Pem arm, which was 6 months longer than that in the Doc/Pem arm. Furthermore, RR and DCR were higher in the G + Doc/Pem arm. However, the frequency of severe toxicities (AEs ≥ grade 3) was significantly higher in the G + Doc/Pem arm (90.9% vs. 45.5%, *p* = 0.032).

Chemotherapy has been suggested to improve the prognosis of patients and is recommended for *EGFR*‐mutant NSCLC based on the findings of a subset analysis of previous phase III studies of EGFR‐TKI.^20^, ^21^ Platinum‐doublet chemotherapies using a third‐generation cytotoxic agent or Pem are recommended for patients <75 years with advanced NSCLC.^22^, ^23^ Meanwhile, in elderly patients aged ≥75 years, carboplatin‐doublet chemotherapies and a third‐generation cytotoxic agent are recommended.^24^, ^25^, ^26^

Although platinum‐based chemotherapies are recommended for healthy elderly patients aged ≥70 years, the number of these patients enrolled in clinical trials is limited.^27^ Many elderly patients do not tolerate chemotherapy well because of progressive reduction in organ function and the presence of comorbidities related to age. Most elderly patients are often not considered eligible for aggressive platinum‐based chemotherapies and are usually treated with only a single cytotoxic agent.^28^ Therefore, we conducted this study using a single cytotoxic agent and targeted elderly patients aged ≥70 years.

The OS and RR in elderly patients with advanced NSCLC on a single third‐generation agent as first‐line treatment were approximately 5.0–14.0 months and 15%–20%, respectively.^6^, ^19^, ^29^ The efficacy of Pem as second‐line treatment was equivalent to that of Doc in patients of various ages with advanced NSCLC.^30^ The Pem and Doc arms had the same median PFS and 1‐year survival rate of 2.9 months and 29.7%, respectively. Small‐scale studies on Pem in elderly patients with advanced NSCLC reported a PFS of 4–5 months and an RR of 15%.^31^, ^32^ The safety profile and efficacy of treatment with Pem was similar to those of treatment with Doc in elderly patients with advanced NSCLC.

Some studies on combination therapy with G and chemotherapy in patients with NSCLC have been conducted. The phases I and II Iressa NSCLC Trial Assessing Combination Treatment (INTACT) study, which focused on *EGFR* mutation‐unknown NSCLC did not demonstrate a benefit in the survival or response to combination therapy. However, combination therapy with G and chemotherapy was safe, with no significant unexpected AEs.^33^, ^34^ In previous studies in elderly patients, a phase II trial on Doc combined with G reported a RR of 40% and a median PFS of 40% (95% CI: 26%–57%) and 6.9 months (95% CI: 3.95–7.8), respectively; this treatment was well‐tolerated.^35^ A recent phase III study reported a significant efficacy of combination therapy with G and chemotherapy for *EGFR*‐mutant advanced NSCLC.^36^ The primary endpoint, PFS, and OS were significantly longer with combination therapy, and a significant difference in RR was observed in the combination arm. The frequency of toxicities of ≥ grade 3 was significantly higher in the combination arm (51% vs. 25%, *p* < 0.001).

Some researchers have reported the efficacy and utility of the continuation of EGFR‐TKIs beyond PD with the addition of cytotoxic agents.^15^, ^37^ However, there was no benefit in continuing G in combination with a platinum‐doublet chemotherapy for *EGFR*‐mutant NSCLC in the IMPRESS study.^38^ This study did not demonstrate the efficacy of continuing G in combination with chemotherapy after disease progression. Therefore, there is a need for clinical trials on combination therapy with the continuation of G and a single‐agent chemotherapy beyond PD for elderly patients with advanced *EGFR*‐mutant NSCLC.

In this study, the efficacy of G + Doc/Pem in terms of RR, PFS, and OS is similar to that of previous studies on DOC and Pem. Rapid exacerbation after one cycle of chemotherapy, such as disease progression, was observed in approximately 20% of the patients (2 of 11 patients) in the Doc/Pem arm.

The need for genotype profiling following rebiopsy in the management of *EGFR*‐mutant NSCLC with acquired resistance to EGFR‐TKIs has recently been suggested. EGFR‐T790M mutation is a key oncogene profile and is responsible for the majority of *EGFR*‐mutant NSCLC with acquired resistance to first‐ and second‐generation EGFR‐TKIs.^39^ Osimertinib was the first EGFR‐TKI recommended for EGFR‐T790M positive NSCLC.^40^, ^41^ For patients who are T790M‐negative at the time of acquired resistance, standard chemotherapy is recommended. However, the best timing to receive chemotherapy in T790M‐negative patients is unclear.

In some studies which have reported the efficacy of continuation therapy with first‐generation EGFR‐TKIs including G and erlotinib beyond PD for advanced *EGFR*‐mutant NSCLC,^17^, ^42^, ^43^, ^44^, ^45^ the continuation of EGFR‐TKIs until symptomatic progression was beneficial for patients with *EGFR*‐mutant NSCLC. Continuing EGFR‐TKIs until symptomatic progression could be an alternative treatment for patients with T790M‐negative NSCLC.

Our limited results suggest that the termination of G therapy and a switch to subsequent chemotherapy after the confirmation of PD based on the RECIST criteria is disadvantageous. Continued G treatment in combination with chemotherapy may be reasonable and beneficial in preventing acute exacerbation due to G withdrawal. In addition, combination chemotherapy may have a synergistic effect.

Rebiopsy is an indispensable technique for deciding the next treatment after progression of initial EGFR‐TKI. Unfortunately, rebiopsy was not planned for in our study and there was no information on histological findings and mutation status before administration of study treatment. Chemotherapy is recommended in cases with unknown cause of initial EGFR‐TKI resistance. Therefore, the results of our trial may show potential efficacy in limited cases in which rebiopsy test cannot be performed or sufficient samples are unavailable for rebiopsy testing analysis. Based on our limited results, combination chemotherapy with continuing gefitinib beyond PD may have potential benefit in limited elderly NSCLC without information on acquired resistance to gefitinib. However, further study is needed to assess the utility of that treatment among those population.

Based on the results of a scheduled interim analysis, this randomized study had important limitations including a poor accrual rate, short follow‐up time, and premature end. Additionally, no molecular information in the enrolled patients with acquired resistance to G was obtained. We analyzed only the registered cases which lacked power to demonstrate our primary endpoint.

Further, our data lacked enough power to demonstrate the clinical utility of continuation of gefitinib as a single agent chemotherapy for second‐line therapy because the study was not completed.

Despite accruing less than half of the planned sample size, this study showed a significantly longer PFS with combination therapy. Our study findings are not generalizable due to the small sample size and incomplete accrual. However, this study provides a basis for considering combination therapy with the continuation of G and chemotherapy beyond PD for elderly patients with advanced *EGFR*‐mutant NSCLC.

In conclusion, combination therapy with the continuation of G and chemotherapy showed a better efficacy but was also associated with an increased toxicity in elderly Japanese patients with *EGFR*‐mutant NSCLC.

## CONFLICT OF INTEREST

This study was supported by the Japan‐Multinational Trial Organization and was funded by AstraZeneca (grant no. ISSIRES0072). K.A received honoraria from AstraZeneca, M.A. received grant from Kyowa Kirin Co. Ltd. for work outside the submitted study. T.Y. and M.M. received honoraria from AstraZeneca, H.Y. received a lecture fee from AstraZeneca and Eli Lilly. S.A. reported personal fees from AstraZeneca, MSD, Eli Lilly, Chugai, Ono, Taiho, Boehringer Ingelheim, Pfizer, Bristol‐Myers Squib, Hisamitsu, and Kyowa Hakko Kirin, and received grants from AstraZeneca, MSD, Eli Lilly, Chugai, Ono, Taiho, Boehringer Ingelheim, Pfizer, F. Hoffmann‐La Roche, and Bristol‐Myers Squibb. All other authors had no conflict of interests to declare.
